# DPP-4 inhibition improves early mortality, β cell function, and adipose tissue inflammation in db/db mice fed a diet containing sucrose and linoleic acid

**DOI:** 10.1186/s13098-016-0138-4

**Published:** 2016-03-01

**Authors:** Jun Shirakawa, Tomoko Okuyama, Mayu Kyohara, Eiko Yoshida, Yu Togashi, Kazuki Tajima, Shunsuke Yamazaki, Mitsuyo Kaji, Megumi Koganei, Hajime Sasaki, Yasuo Terauchi

**Affiliations:** Department of Endocrinology and Metabolism, Graduate School of Medicine, Yokohama-City University, 3-9 Fukuura, Kanazawa-ku, Yokohama, 236-0004 Japan; Food Science Research Laboratories, R&D Division, Meiji Co., Ltd., Odawara, Japan; Department of Nutritional and Life Sciences, Kanagawa Institute of Technology, Atsugi, Japan

**Keywords:** Type 2 diabetes, DPP-4 inhibitor, Life span, Insulin resistance, Pancreatic β cell, Adipose tissue

## Abstract

**Background:**

Diabetes therapy that not only lowers glucose levels but also lengthens life spans is required. We previously demonstrated that DPP-4 inhibition ameliorated β cell apoptosis and adipose tissue inflammation in β cell-specific glucokinase haploinsufficient mice fed a diet containing a combination of sucrose and linoleic acid (SL).

**Methods:**

In this study, we investigated the effects of DPP-4 inhibition in obese diabetic db/db mice fed an SL diet or a control diet containing sucrose and oleic acid (SO). We also examined the effects of DPP-4 inhibition in IRS-1-deficient mice fed an SL or SO diet as a model of insulin resistance.

**Results:**

DPP-4 inhibition efficiently increases the active GLP-1 levels in db/db mice. Unexpectedly, the SL diet, but not the SO diet, markedly increases mortality in the db/db mice. DPP-4 inhibition reduces the early lethality in SL-fed db/db mice. DPP-4 inhibition improves glucose tolerance, β cell function, and adipose tissue inflammation in db/db mice fed either diet. No significant changes in glycemic control or β cell mass were observed in any of the IRS-1-deficient mouse groups.

**Conclusions:**

A diet containing a combination of sucrose and linoleic acid causes early lethality in obese diabetic db/db mice, but not in lean and insulin resistant IRS-1 knockout mice. DPP-4 inhibition has protective effects against the diet-induced lethality in db/db mice.

**Electronic supplementary material:**

The online version of this article (doi:10.1186/s13098-016-0138-4) contains supplementary material, which is available to authorized users.

## Background

Accumulating evidence suggests that diabetic patients have a decreased life span, compared with non-diabetic subjects [1, 2]. Therefore, promoting longevity is one of the definitive goals for the cure and care of diabetes. The activation of insulin signaling is a major approach for blood glucose-lowering therapy, while the loss of insulin receptor-mediated signaling has resulted in extensions of the life spans in several models [3, 4]. Dietary sugar and fat intake influences not only metabolism in various metabolic tissues, but also aging [5]. However, the effects of dietary fatty acids or diabetes therapy on life span in diabetic patients remain obscure.

Palmitic acid, oleic acid, and linoleic acid are the three major fatty acids among plasma lipids [6]. We previously investigated diet-induced metabolic changes in β cell-specific glucokinase haploinsufficient (βGck^+/−^) diabetic mice fed a diet containing a combination of sucrose and oleic acid (SO) or sucrose and linoleic acid (SL) [7, 8]. SL induced β cell apoptosis and adipose tissue inflammation in βGck^+/−^ mice. βGck^+/−^ mice exhibit impaired insulin secretion in response to glucose but have normal insulin sensitivity. In Zucker fatty (fa/fa) rat, a model of obesity and insulin resistance, SL diet showed β cell failure, enhanced macrophage infiltration in adipose tissue, and an elevated plasma tumor necrosis factor-α concentration compared with SO diet [9]. These results inspired us to investigate the impacts of SL and SO diets on metabolic tissues in obese diabetes model mice. We also reported that treatment with a dipeptidyl peptidase-4 (DPP-4) inhibitor ameliorated SL diet-induced β cell apoptosis and adipose tissue inflammation in βGck^+/−^ mice [7, 8]. DPP-4 inhibitors induced increases in active incretins (GLP-1 and GIP) and other circulating peptides by slowing enzymatic cleavage, thereby enhancing incretin-induced glycemic control. A number of pre-clinical studies have also suggested the possibility that GLP-1 receptor agonists and DPP-4 inhibitors exhibit pleiotropic metabolic actions, such as cardioprotection [10, 11]. A meta-analysis of randomized clinical trials suggested that DPP-4 inhibitors reduced cardiovascular events and all-cause mortality in patients with type 2 diabetes with a mean follow-up period of 44.1 weeks [12]. In an aged, high-fat-diet-induced obesity mouse model, the survival rates were improved by chronic DPP4 inhibition [13]. However, the EXAMINE and SAVOR-TIMI 53 phase III/IV trials showed no significant differences in cardiovascular events between DPP-4 inhibitors and a placebo in type 2 diabetic patients [14, 15]. Hence, the effect of DPP-4 inhibition on longevity has remained controversial.

Obese diabetic db/db mice are morbidly obese and exhibit severe insulin resistance, hyperglycemia, and diabetic complications [16, 17]. High-fat-diet-loading reportedly shortens the life span of db/db mice [18]. In the present study, we examined the effects of DPP-4 inhibitors on glucose tolerance, β cell loss, and adipose tissue inflammation in obese diabetic db/db mice fed an SL or SO diet. Although both of these diets contained similar ratios of fat and saturated fatty acids, the SL diet increased the mortality rate of db/db mice. Interestingly, DPP-4 inhibition enabled a nearly complete restoration of the mortality rate in SL-fed db/db mice. We also assessed the effects of DPP-4 inhibition in IRS-1 deficient mice fed an SL or SO diet as a model of insulin resistance without obesity.

## Methods

### Animals and animal care

Animal study was carried out in strict accordance with the recommendations in the Guide for the Care and Use of Laboratory Animals of the Yokohama City University. The protocol was approved by the Yokohama City University Institutional Animal Care and Use Committee (IACUC) (Permit Number: 11-29, F-A-13-043). All experiments were performed under appropriate anesthesia, and all efforts were made to minimize suffering.

Littermate db/db and db/+ mice aged 7 weeks were purchased from Charles River Japan (Yokohama, Japan). We backcrossed IRS-1^−/−^ mice with C57Bl/6J mice more than 10 times [19]. The mice were fed a standard-chow diet (MF, Oriental Yeast, Japan) until 8 weeks of age and then were given free access to the experimental diets. All the experiments were conducted on male littermates. Animal housing rooms were maintained at a constant room temperature (25 °C) and a 12-h light (7:00 a.m.)/dark (7:00 p.m.) cycle. In the survival study, mice were monitored two times a day for clinical signs as described in the Yokohama City University Institutional Animal Care and Use Committee (IACUC) policy to categorize animals as morbid or moribund. Mice were sacrificed when distress was apparent as defined by our IACUC approved animal protocol (hunched posture, reduced activity, altered respiratory pattern such as abdominal breathing, severe weight loss, or inability to stand). Animals judged to be moribund were euthanatized with an anesthesia (mixture of medetomidine hydrochloride, midazolam, and butorphanol tartrate) and counted as lethality. At the end of these experimental procedures, the remaining mice were also euthanized with an anesthetics.

### Diets

The compositions of the SO and SL diets are described in Additional file 1: Table S1 [7, 8]. The fat component of the SO and SL diets was derived from safflower oil and high-oleic sunflower oil blended with perilla oil, respectively. The two diets were identical except for the type of fat used: oleic acid was used in the SO diet, and linoleic acid was used in the SL diet. Both diets contained similar amounts of palmitic acids. The experimental diets were freshly prepared weekly. A DPP-4 inhibitor des-fluoro-sitagliptin (DFS) was administered orally by premixing with SO or SL to a concentration of 0.4 % [20]. Another DPP-4 inhibitor, MK-0626, was administered orally by premixing with SO or SL to a concentration of 0.0045 % [21]. Both the DFS and the MK-0626 used in this study were provided by Merck & Co., Inc.

### Biochemical parameters

The blood glucose levels were determined using a Glutest Neo Super, Glutest Mint (Sanwa Chemical Co., Japan) or Glucose Assay Kit (BioVision, CA). Serum insulin levels and the triglyceride content in the liver were determined using an insulin kit (Morinaga, Japan) and the Determiner-L TG II kit (Wako Pure Chemical Industries, Japan), respectively. The levels of plasma alanine aminotransferase, free fatty acid, total cholesterol, and triglyceride were assayed using enzymatic methods (Wako Pure Chemical Industries). Serum DPP-4 activity was measured using a DPP4 Activity Assay Kit (Bio Vision, CA). Active GLP-1 was assayed using a Glucagon-Like Peptide-1 (Active) ELISA Kit (Millipore, MA). An insulin tolerance test (ITT) was performed by intraperitoneally injecting mice with human insulin (1.5 mU/g body weight). An oral glucose tolerance test (OGTT) was performed by withholding all food from the mice for more than 18 h and then orally loading the mice with glucose (1.5 mg/g body weight).

### Histological analysis

Formalin-fixed, paraffin-embedded pancreas or adipose tissue sections were immunostained with antibodies to insulin (Santa Cruz, sc-9168), glucagon (Abcam, ab10988), or F4/80 (Serotec, MCA497). Biotinylated secondary antibodies, a VECTASTAIN elite ABC kit, and a DAB substrate kit (VECTOR) were used to examine the sections using bright-field microscopy, and Alexa Fluor 488- and 555-conjugated secondary antibodies (Invitrogen) were used for fluorescence microscopy. All the images were acquired using a BZ-9000 microscope (Keyence) or a Carl Zeiss LSM 510 confocal laser-scanning microscope. The percent area of the pancreatic tissue occupied by the β cells was calculated using BIOREVO software (Keyence), as described previously [22]. More than five tissue sections from each animal, including representative sections of each tissue region, were analyzed.

### Real-Time PCR

Tissue specimens were preserved in RNAlater reagent (QIAGEN) until the isolation of the total RNA. Total RNA was isolated from the epididymal fat using an RNeasy Lipid tissue kit (QIAGEN). cDNA was prepared using the TaqMan reverse transcriptase kit (Applied Biosystems) and was subjected to quantitative PCR using TaqMan Gene Expression Assays (7900 real-time PCR system; Applied Biosystems) with THUNDERBIRD qPCR Master Mix (TOYOBO). Transcription of each gene was detected using TaqMan Gene Expression Assays (Thermo Fisher Scientific Inc.): F4/80 (Adgre1, Mm00802529_m1), CD11c (Itgax, Mm00498698_m1), TNF-α (Tnf, Mm00443258_m1), MCP-1 (Ccl2, Mm00441242_m1), and PAI-1 (Serpine2, Mm00436753_m1). The data was normalized according to the β-actin (Actb, Mm02619580_g1). Each quantitative reaction was performed in duplicate.

### Statistical analyses

All the data are expressed as the mean ± S.E. and were analyzed using an ANOVA. Differences were considered significant if the *P* value was <0.05 (*, †).

## Results

### A single oral dose of DPP-4 inhibitors sufficiently suppressed DPP-4 activity in db/db mice

**Fig. 1:**
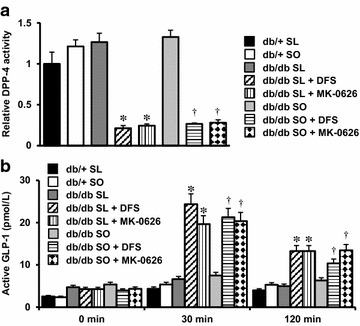
Changes in serum DPP-4 activity and active GLP-1 concentrations in db/+ mice and db/db mice during an oral meal tolerance test. The experiments were performed in db/+ or db/db mice fed an SL diet, an SO diet, or a diet containing the DPP-4 inhibitor 0.4 % des-fluoro-sitagliptin or 0.0045 % MK-0626. **a** Serum DPP-4 activity was measured in mice fed the indicated diets ad libitum (n = 5). *P < 0.05 vs. db/db SL. ^†^P < 0.05 vs. db/db SO. **b** Serum active GLP-1 concentration at 0 min (fasted > 20 h), 30, and 120 min after the oral administration of each diet test meal (12 mg/g body weight) in db/+ mice and db/db mice that had been fed either the SO or SL diet (n = 3–4). To obtain a sufficient amount of whole blood to measure the biologically active form of GLP-1, blood was collected from the inferior vena cava with a DPP-4 inhibitor (Millipore) at the time points indicated. *P < 0.05 vs. db/db SL. ^†^P < 0.05 vs. db/db SO

To assess the effects of DPP-4 inhibitor in db/db mice fed an SL or SO diet (Additional file 1: Table S1), we performed an oral meal tolerance test (12 mg/g body weight) in 8-week-old db/+ or db/db mice. The DPP-4 inhibitors des-fluoro-sitagliptin (DFS) and MK-0626 were separately premixed with SO or SL at a concentration of 0.4 or 0.0045 %, respectively. DPP-4 is thought to be an adipokine that is released from adipose tissue at a higher level in obese individuals [23]. However, the DPP-4 activities were similar between the db/+ mice and the db/db mice fed an SO or SL diet (Fig. 1a). DFS and MK-0626 similarly inhibited the serum DPP-4 activity by approximately 80 % in db/db mice fed an SL or SO diet (Fig. 1a). We next measured the serum active GLP-1 concentration after oral loading with an SO or SL meal (12 mg/g body weight) in the presence or absence of a DPP-4 inhibitor in standard-chow diet-fed db/+ or db/db mice. The results showed no significant differences in serum active GLP-1 concentrations between the SO-fed and the SL-fed db/+ mice or db/db mice at 0, 30, or 120 min after feeding (Fig. 1b). The serum active GLP-1 concentrations were significantly increased by DPP-4 inhibition with DFS or MK-0626 in db/db mice fed an SO or SL diet (Fig. 1b). Thus, DFS and MK-0626 efficiently inhibited the DPP-4 activity and increased the active GLP-1 levels in db/db mice. We previously reported that DFS improved β cell ER stress, adipose tissue inflammation, and hepatic steatosis in lean diabetic βGck^+/−^ mice [7, 8]. Hence, we used DFS as the DPP-4 inhibitor for the db/db mouse model in this study.

### Sucrose- and linoleic acid-diet-induced early mortality in db/db mice and reduction in lethality by DPP-4 inhibition

**Fig. 2:**
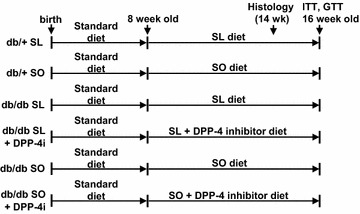
Experimental protocol. Both the db/+ and db/db mice were fed a standard chow diet until 8 weeks of age and were then given free access to the experimental diets. Experiments were performed on db/+ and db/db mice after 8 weeks on the SL diet, SO diet, SL + DPP-4 inhibitor (0.4 % des-fluoro-sitagliptin) diet, or SO + DPP-4 inhibitor (0.4 % des-fluoro-sitagliptin) diet

**Fig. 3:**
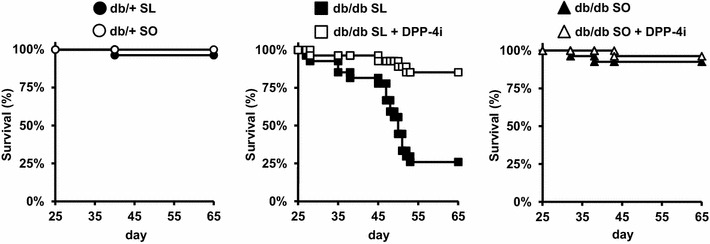
SL-diet-induced early mortality in db/db mice and DPP-4 inhibition reduced lethality. Survival rates of indicated mice (n = 27). The db/+ and db/db mice were fed the SL diet, SO diet, SL + DPP-4 inhibitor diet, or SO + DPP-4 inhibitor diet, as described in Fig. 2

Db/+ mice and db/db mice fed an SL diet or an isocaloric SO diet for 8 weeks were evaluated for glucose tolerance and phenotypic changes in metabolic tissues (Fig. 2). To evaluate the effect of a DPP-4 inhibitor as a treatment for diet-induced metabolic dysfunction in obese diabetic mice with severe insulin resistance, we also performed an 8-week study comparing db/db mice fed a diet consisting of SL or SO plus DPP-4 inhibitor (Fig. 2). Unexpectedly, early lethality at 1–2 months after the start of the experiments was observed in the SL-fed db/db mice, but not in the SO-fed mice or db/db mice (Fig. 3). Over 70 % of the db/db mice died after 5–8 weeks of SL-loading. In contrast, db/db mice fed an SL + DPP-4 inhibitor diet exhibited more than 80 % survival at the end of the experiment period (Fig. 3). We performed three independent 8-week meal loading tests and combined these results (Additional file 2: Figure S1). No apparent signs of injury or infection were observed in the dead SL-fed db/db mice. We also confirmed that none of the db/db mice fed a standard chow diet died before 16 weeks of age.

### No significant changes in insulin sensitivity and glucose tolerance between SL-fed db/db mice and SO-fed db/db mice and improvement induced by DPP-4 inhibitor

**Fig. 4:**
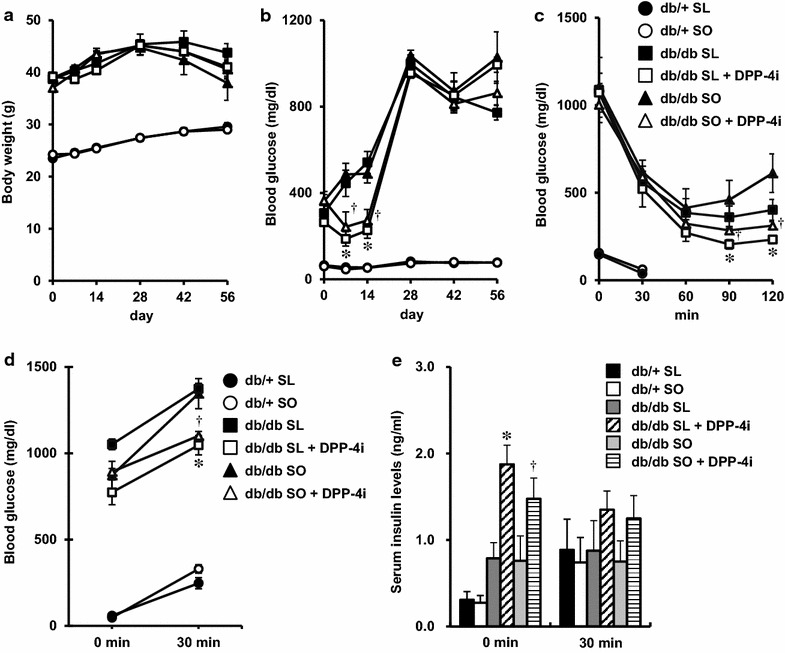
DPP-4 inhibitor improved insulin sensitivity and glucose tolerance in SL- or SO-fed db/db mice. The experiments were performed in 16-week-old mice, as shown in Fig. 2 (n = 6–11). **a** Body weight gain. **b** Blood glucose levels. *P < 0.05 vs. db/db SL. ^†^P < 0.05 vs. db/db SO. **c** Blood glucose levels during insulin tolerance test (*ITT*). *P < 0.05 vs. db/db SL. ^†^P < 0.05 vs. db/db SO. **d** Blood glucose levels during oral glucose tolerance test (*OGTT*). *P < 0.05 vs. db/db SL. ^†^P < 0.05 vs. db/db SO. **e** Serum insulin levels during OGTT. *P < 0.05 vs. db/db SL. ^†^P < 0.05 vs. db/db SO

Compared with the db/+ mice, the db/db mice showed an increased body weight, liver weight, epididymal fat weight, blood glucose level, insulin resistance, and glucose intolerance after feeding with either the SL or SO diet (Fig. 4a–d; Additional file 3: Figure S2). No significant differences in body weight, liver weight, blood glucose level, insulin sensitivity, glucose tolerance, or insulin secretion after glucose loading were observed between the SO group and the SL group for either genotype (Fig. 4a–e; Additional file 3: Figure S2). Treatment with the DPP-4 inhibitor had no significant effect on body weight gain, liver weight, or epididymal fat weight in db/db mice fed an SL or SO diet (Fig. 4a; Additional file 3: Figure S2). The blood glucose levels were decreased by the addition of the DPP-4 inhibitor to the diets until the second week of the experiment in both groups (Fig. 4b). After the manifestation of severe hyperglycemia in the db/db mice fed an SL or SO diet, treatment with the DPP-4 inhibitor was no longer capable of reducing the blood glucose levels (Fig. 4b). However, DPP-4 inhibition significantly decreased the blood glucose levels at 90 or 120 min in an insulin tolerance test and at 30 min in an oral glucose tolerance test in db/db mice after SL or SO feeding for 8 weeks (Fig. 4c, d). The blood glucose levels at 60, 90, 120 min during glucose tolerance test showed values over the limit of detection of the glucometers in db/db mice fed an SL or SO diet, but not in DPP-4 inhibitor-treated db/db mice. Furthermore, the fasting serum insulin levels were significantly elevated in DPP-4 inhibitor-treated db/db mice in both diet groups (Fig. 4e). These results indicated that the DPP-4 inhibitor provided a slight but significant improvement in insulin sensitivity and glucose tolerance, even in obese hyperglycemic db/db mice fed an SL or SO diet.

### DPP-4 inhibitor protected against β cell failure in db/db mice fed an SL or SO diet

**Fig. 5:**
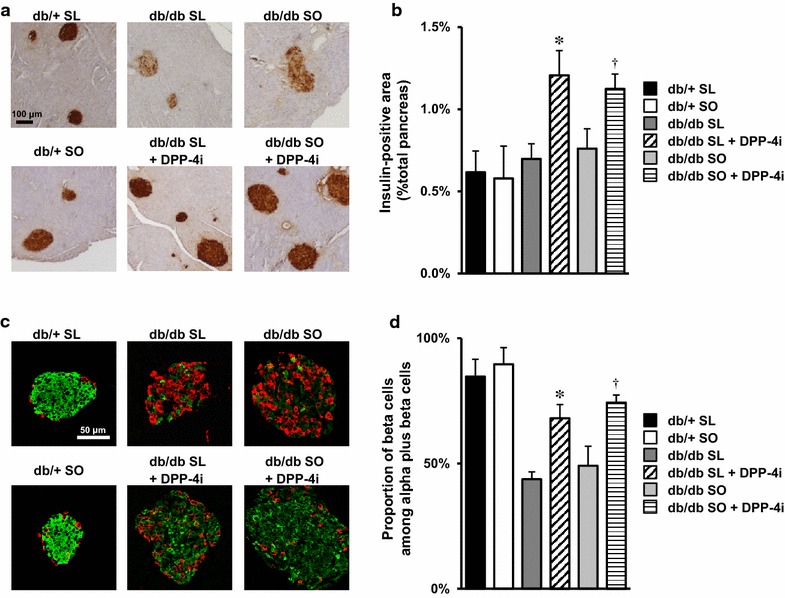
DPP-4 inhibitor increased β cell mass and ameliorated islet morphology in SL- or SO-fed db/db mice. The experiments were performed in 14-week-old mice, as shown in Fig. 2. **a** Representative pancreatic sections stained with antibodies for insulin (*brown*) are shown. The *scale bar* represents 100 μm. **b** β cell mass (n = 6–8). The β cell area is shown as a proportion of the area of the entire pancreas. **c** Representative pancreatic sections stained with antibodies for insulin (*green*) and glucagon (*red*) are shown. The *scale bar* represents 50 μm. **d** Quantification of β cell mass as a proportion of the total α-cell-plus-β-cell mass in the islet (n = 6). *P < 0.05 vs. db/db SL. ^†^P < 0.05 vs. db/db SO

SL significantly reduced the β cell mass and the β cell proportion in islet cells through a greater increase in apoptosis, compared with that induced by SO, in lean diabetic βGck^+/−^ but not wild-type mice [7]. Obese diabetic db/db mice fed a normal chow diet showed a transient increase in β cell mass and proliferation from 1 to 3 months of age, followed by a subsequent decrease with further aging [24]. To analyze the effects of the SL and SO diets on β cell loss in db/db mice, we investigated the β cell mass and the β cell proportion in islet cells in SL- or SO-diet-fed 14-week-old db/db mice (Fig. 2). No significant changes in β cell mass or β cell proportion were observed between the SL and SO groups in both db/+ and db/db mice (Fig. 5a–d). An abnormal distribution of β cells in the islets was observed in db/db mice fed an SL or SO diet, compared with db/+ mice (Fig. 5a, c). The β cell mass and islet morphology in SL- or SO-fed db/db mice were similar to those observed in normal chow-fed db/db mice of the same age (data not shown). These results suggested that the influence of the difference in the SL and SO diets was less than that of the intrinsic mechanism responsible for β cell loss in db/db mice.

We previously demonstrated that DPP-4 inhibition ameliorated β cell ER stress and apoptosis in SL-fed βGck^+/−^ mice, and the GLP-1 receptor agonist liraglutide protected against reductions in β cells in neonatal βGck^−/−^ mice independent of insulin secretion [7, 25]. As expected, the treatment of SL- and SO-fed-db/db mice with a DPP-4 inhibitor for 8 weeks produced a significant increase in β cell mass (Fig. 5b) and the relative β cell mass as a proportion of the total α-cell-plus-β-cell mass (Fig. 5d); furthermore, the abnormal distribution of pancreatic α cells was also corrected (Fig. 5c). The intensity of the fluorescence signals of insulin was also augmented by DPP-4 inhibition in db/db mice (Fig. 5c). Thus, DPP-4 inhibition ameliorated β cell failure in db/db mice regardless of whether the mice were fed an SL or SO diet.

### Inflamed adipose tissue was mitigated by DPP-4 inhibition in db/db mice fed an SL or SO diet

**Fig. 6:**
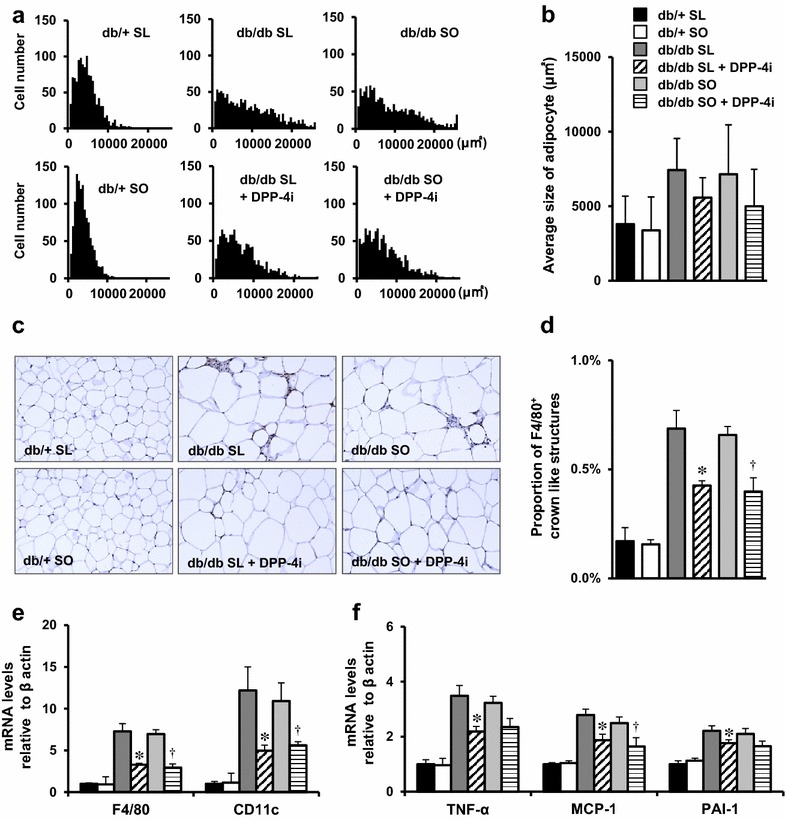
Inflamed adipose tissue was improved by DPP-4 inhibition in db/db mice fed an SL or SO diet. The experiments were performed in 14-week-old mice, as shown in Fig. 2. **a** Representative histogram of adipocyte size in epididymal fat. **b** Average size of adipocyte from indicated mice (n = 5). **c** Epididymal fat tissue was stained with anti-F4/80 antibody. **d** The number of F4/80^+^ crown-like structures (*CLSs*) was counted as described in the Methods (n = 5). *P < 0.05 vs. db/db SL. ^†^P < 0.05 vs. db/db SO. **e**, **f** Assessment of the levels of expression of the indicated mRNAs in epididymal fat as determined using real-time quantitative RT-PCR and normalization to the β-actin mRNA level (n = 5). *P < 0.05 vs. db/db SL. ^†^P < 0.05 vs. db/db SO

Inflammation induced by the infiltration of macrophages or other hemocytes into adipose tissue contributes to obesity-related insulin resistance in db/db mice [26]. Compared with SO, SL increased CD11c^+^ M1 macrophage infiltration into visceral adipose tissue in βGck^+/−^ mice [8]. Therefore, we analyzed the visceral adipose tissues of SL- or SO-fed db/db mice. The adipocyte area in db/db mice fed an SL or SO diet was larger than that in SL or SO diet-fed db/+ mice (Fig. 6a, b). An immunohistochemical analysis revealed that the proportion of F4/80^+^ crown-like structures (CLSs) in adipocytes was also increased in db/db mice compared with db/+ mice, in both the SL and SO groups (Fig. 6c, d). The mRNA expression levels of F4/80, CD11c, TNF-α, MCP-1, and PAI-1 were significantly higher among the db/db mice fed an SL or SO diet (Fig. 6e, f). No significant changes in the adipocyte area, the number of F4/80^+^ CLSs, inflammatory gene expressions, or serum lipid parameters were observed between the SL and SO groups in the db/db or db/+ mice (Fig. 6a–f; Additional file 4: Figure S3). These results indicated that adipose tissue inflammation was not caused by the difference in the compositions of the diets that were used to feed the mice. In fact, we also confirmed that db/db mice of the same age that were fed a standard diet showed F4/80^+^ CLSs and inflammatory gene expressions in adipocytes to a similar degree as that observed in db/db mice fed an SL or SO diet (data not shown).

In SL-fed βGck^+/−^ mice, DPP-4 inhibition averts adipose tissue inflammation by reducing the infiltration of CD11c^+^ M1 macrophages and CD8^+^ T cells [8]. To evaluate the use of a DPP-4 inhibitor as a treatment for adipose tissue inflammation in obese diabetic db/db mice with SL or SO, we examined visceral adipose tissue inflammation in DPP-4 inhibitor-treated db/db mice. The epididymal fat weight and lipid parameters in the SL- or SO-fed db/db mice were not affected by the addition of a DPP-4 inhibitor to the diet (Additional files 3 and 4: Figures S2 and S3). The adipocyte size tended to be decreased by the treatment with DPP-4 inhibitor in these mice (Fig. 6a, b). DPP-4 inhibitor attenuated the proportion of F4/80^+^ CLSs and macrophage-related inflammatory gene expressions in the adipocytes of db/db mice in both diet groups (Fig. 6c–f).

We previously demonstrated that treatment with a DPP-4 inhibitor improved hepatic steatosis in both SL-fed and SO-fed βGck^+/−^ mice [8]. However, no significant differences in the hepatic triglyceride contents were observed in SL- or SO-fed db/db mice in the presence or absence of a DPP-4 inhibitor (Additional file 5: Figure S4).

### No significant effects of an SL or SO diet and DPP-4 inhibition in insulin-resistant IRS-1-deficient mice

**Fig. 7:**
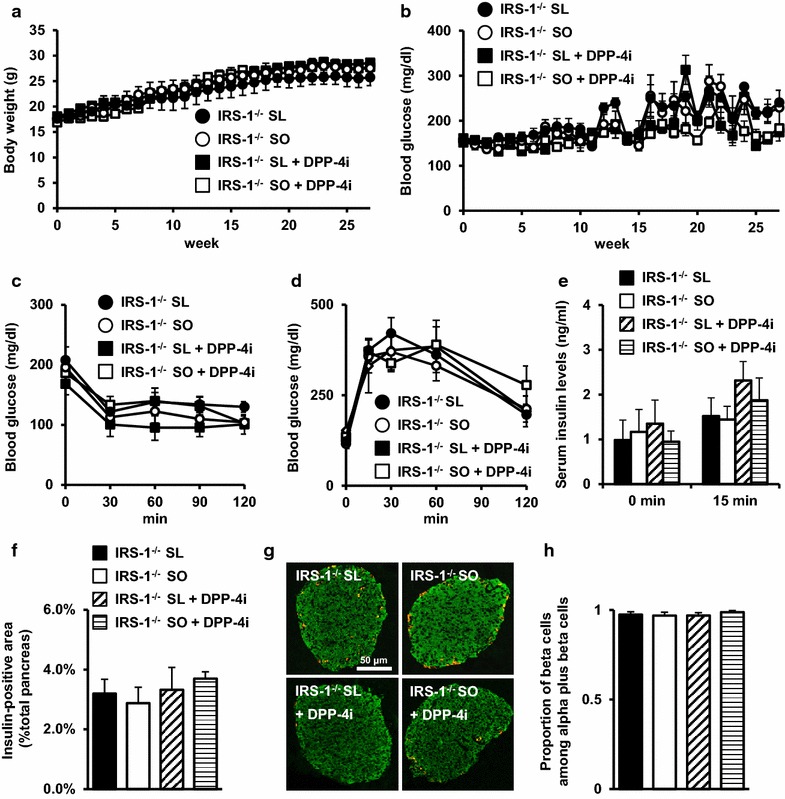
No significant effects of SL or SO diet and DPP-4 inhibitor in insulin-resistant IRS-1 deficient mice. The experiments were performed in IRS-1 deficient (IRS-1^−/−^) mice after 27 weeks on an SL diet, SO diet, SL + DPP-4 inhibitor (0.4 % des-fluoro-sitagliptin) diet, or SO + DPP-4 inhibitor (0.4 % des-fluoro-sitagliptin) diet. **a** Body weight gain (n = 6). **b** Blood glucose levels (n = 6). **c** Blood glucose levels during insulin tolerance test (*ITT*) (n = 6). **d** Blood glucose levels during oral glucose tolerance test (OGTT) (n = 6). **e** Serum insulin levels during OGTT (n = 6). **f** β cell mass (n = 6–8). The β cell area is shown as a proportion of the area of the entire pancreas. **g** Representative pancreatic sections stained with antibodies for insulin (*green*) and glucagon (*red*) are shown. The *scale bar* represent 50 μl. **h** Quantification of β cell mass as a proportion of the total α-cell-plus-β-cell mass in the islet (n = 6)

In db/+ or db/db mice, differential effects of an SL or SO diet on glucose metabolism were not observed (Figs. 4, 5, 6) [8]. An SL diet aggravated β cell function and adipose tissue inflammation in lean βGck^+/−^ mice with impaired insulin secretion in response to glucose and normal insulin resistance, compared with an SO diet [7, 8]. An SL diet, but not an SO diet, shortened the life spans of obese diabetic db/db mice with severe insulin resistance (Fig. 3). To assess the impact of an SL or SO diet under an insulin-resistant state, we used an SL or SO diet with or without a DPP-4 inhibitor to feed wild-type (WT) mice and insulin receptor substrate (IRS)-1-deficient (IRS-1^−/−^) mice for 27 weeks. The IRS-1^−/−^ mice showed normal glucose tolerance, peripheral insulin resistance (especially in skeletal muscle), compensatory β cell mass hyperplasia, and growth retardation [19]. The body weight and blood glucose levels were not affected by the dietary composition or DPP-4 inhibition in IRS-1^−/−^ mice (Fig. 7a, b). An SL or SO diet containing a DPP-4 inhibitor had no significant effects on insulin sensitivity, glucose tolerance, or insulin secretion in response to glucose gavage (Fig. 7c–e). No significant changes in β cell mass or β cell proportion were observed between the SL and SO groups with or without a DPP-4 inhibitor in IRS-1^−/−^ mice (Fig. 7f–h). Accordingly, the aggravating effects of an SL diet might depend on hyperglycemia or β cell dysfunction, but not insulin resistance, in mice.

## Discussion

Here, we established a model in which a diet rich in sucrose and linoleic acid induced early mortality in obese diabetic db/db mice. Db/db mice are a well-known model of obesity, diabetes, insulin resistance, β cell failure, adipose tissue inflammation, diabetic complications including heart failure, and so on [16, 17]. The extension of the life span is the primary goal of medical therapy for any disease. Therefore, our model should be a good model for studying the diet-induced exacerbation of life-threatening disease. Short-lived SL-fed db/db mice, however, demonstrated glucose intolerance, insulin resistance, β cell failure, and adipose tissue inflammation to degrees similar to those of SO-fed db/db mice. Hence, the SL-induced shortening of the life span in db/db mice was thought to be caused by unidentified mechanisms.

Our previous study demonstrated that an SL diet induced β cell apoptosis and adipose tissue inflammation in βGck^+/−^ mice [7, 8]. βGck^+/−^ mice manifested post-prandial hyperglycemia caused by impaired glucose-induced insulin secretion and failed to proliferate β cells in response to insulin resistance by high-fat diet loading [27, 28]. Glucokinase in β cells also plays a crucial role in the protection of β cells against ER stress-induced apoptosis through IRS-2 dependent and independent pathways [29]. In db/db mice, glucokinase activation by a glucokinase activator improved the glycemic profiles [30]. Therefore, glucokinase-mediated signals might be intact in db/db mice, and the function of β cell glucokinase, but not insulin resistance, could contribute to SL-induced β cell failure and adipose tissue inflammation. Insulin resistant IRS-1^−/−^ mice demonstrated insulin secretory defects, hyperplastic islets, hyperinsulinemia, and normoglycemia [19, 31]. The results of IRS-1^−/−^ mice fed an SL or SO in this study showed no significant changes in the metabolic phenotypes or β cell mass, indicating that insulin resistance was not the cause of β cell failure. Since db/db mice become obese and uncontrollable hyperglycemia due to the spontaneous mutation of leptin receptor (LepR/ObR), the β cell failure and inflamed adipose tissue might be derived from the dysfunction of LepR/ObR-signaling, and not from dietary components in db/db mice fed an SL or SO diet.

Then, what is the cause of the early mortality in SL-fed db/db mice? Due to strict ethical regulations involving research animal studies, we are required to euthanize animals when they become sick or injured. The most common cause of death was therefore euthanization due to unknown disorders. The severity of myocardial ischemia and reperfusion injury, the development of congestive heart failure, and the mortality after myocardial ischemia were markedly exacerbated in db/db diabetic mice [32]. In diabetic db/db mice, the monocyte/endothelial interaction was accelerated by an increase in the production of 12/15 lipoxygenase [33]. Linoleic acid is converted to arachidonic acid in many animal tissues, and a linoleic acid-rich SL-diet has been reported to increase the tissue content of arachidonic acid [7], which is a precursor of prostaglandins, HETEs, and leukotrienes. Of note, cardiac necrosis was reportedly induced by the excessive loading of linoleic acid in STZ-induced hyperglycemic rats [34, 35]. However, a systematic review and meta-analysis of prospective cohort studies indicated that the linoleic acid intake is inversely associated with the cardiovascular heart disease risk in a dose–response manner [36]. We also preliminarily evaluated the heart histology of SL- or SO-fed db/db mice in this study, but no apparent differences in ischemia or fibrotic lesions were observed (Additional file 6: Figure S5). The deficiency of LepR/ObR signaling in SL-fed db/db mice might participate in the early mortality, because insulin resistant IRS-1 knockout mice fed an SO or SL diet showed no abnormality in survival rate compared with those fed normal chow. Accordingly, further research is needed to clarify the mechanisms of SL-induced death in db/db mice.

The results of this study also showed that DPP-4 inhibition protected against an SL-diet-induced shortened life span in db/db mice. DPP-4 inhibition also ameliorated glycemic control, β cell loss, and adipose tissue inflammation in db/db mice fed either an SL or SO diet. We previously reported that DPP-4 inhibition improved β cell loss and adipose tissue inflammation without inducing any changes in fatty acid contents in the tissues of SL-fed diabetic mice [7, 8]. So, the protective effect of DPP-4 inhibition on life span in db/db mice was independent of alterations in fatty acid contents in tissues. However, although a preliminary analyses suggested no differences in food intake among all groups, the differences in fatty acid intake or absorption caused by diets or DPP-4 inhibition might exist and function as contributing factors. Evidence has suggested that postprandial hyperglycemia is an independent risk factor for all causes of death and cardiovascular disease [37]. The suppression of glucose spikes after glucose loading induced by DPP-4 inhibition in db/db mice might be one explanation of its protective effects on longevity. The improvement of cardiac function after myocardial infarction through a combination of DPP-4 inhibition and G-CSF administration has been reported, and liraglutide was able to confer cardioprotection and a survival advantage after myocardial infarction [38, 39]. In addition to incretins, increased SDF-1α or BNP by DPP-4 inhibition might contribute to the protection or the regeneration of cardiomyocytes in this study [40]. Interestingly, DPP-4 itself is reportedly an obesity-related adipokine that might worsen insulin resistance [23]. In this study, however, both SL- and SO-fed db/db mice showed similar DPP-4 activities and circulating active GLP-1 levels. Furthermore, the efficiency of DPP-4 inhibition as measured by the DPP-4 activity and serum active GLP-1 levels showed no significant differences between an SL diet and an SO diet in db/db mice. Collectively, these results suggest that an insufficiency of DPP-4 activity was not responsible for the shortened life span of db/db mice fed an SL diet and increase of SDF-1α, BNP, or other DPP-4 target factors by DPP-4 inhibition might be involved in longevity of those mice. Treatment with the GLP-1 receptor agonist liraglutide improved the decrease in the β cell mass and fatty liver independent of insulin secretion in β cell-specific glucokinase homozygous knockout mice but failed to prolong survival, and all the mice died within 1 week [25]. The incretin-induced increment in β cell mass and the prolonged longevity of db/db mice might not be related. Recently, a report suggested that adipose tissue inflammation increases hepatic acetyl CoA and causes hepatic insulin resistance through inflammatory cytokine production in high-fat-diet-fed rats [41]. We demonstrated that DPP-4 inhibition reduced adipose tissue inflammation and inflammatory cytokine expression, even in db/db mice. Since the adipose tissue is the largest endocrine organ in the body, an investigation of the inter-organ network involving the adipose tissue before and after DPP-4 inhibition could be useful for determining the effects of DPP-4 inhibition on life span in our model.

The dose of des-fluoro-sitagliptin and MK-0626, DPP-4 inhibitors used in this study, may be higher than a body weight-based dose of DPP-4 inhibitors in the clinical practice. There might be pharmacodynamic differences in the absorption and persistent effects of a DPP-4 inhibitor in this study because DPP-4 inhibitors are orally administrated, not mixed in diet, in the clinical practice and the absorbance of lipophilic drugs are elevated by bile especially in the postprandial period. Thus, consideration should be given to the possibility that the time- and dose-dependent effects of DPP-4 inhibitor contributed to the outcome.

In addition to db/db mice, Zucker diabetic fatty fa/fa (ZDF) rats, harboring a missense mutation (fatty, fa) in the leptin receptor gene (Lepr/ObR), are also widely used as a model of obese type 2 diabetes. In previous reports, the DPP-4 inhibitor, sitagliptin, prevented β cell dysfunction with anti-apoptotic, anti-inflammatory, anti-oxidant, pro-angiogenic and pro-proliferative effects in ZDF rats [42, 43]. Sitagliptin also corrected the hyperglycemia, hyperlipidemia, inflammation, and hypertension in ZDF rats [43]. Furthermore, the treatment of ZDF rats with sitagliptin also ameliorated nitrosative stress, inflammation and apoptosis in retinal cells, and prevented diabetic nephropathy progression through anti-inflammatory and anti-apoptotic properties [44–46]. These protective actions of sitagliptin have important implications for a better understanding how DPP-4 inhibition prevented the early mortality in an obese diabetes model.

Numerous studies have demonstrated that diabetes and obesity are related with decreased longevity in human [1, 47]. Prolonging the life span is the eventual aim of the treatment of diabetes and obesity. Our diet-induced db/db mice are a good model for the study of survival and longevity in obese and diabetic subjects. The effects of DPP-4 inhibition on life span have been obscure [13–15, 19]. The effect of DPP4 inhibition on weight is neutral, while most other hypoglycemic agents increase weight gain [48]. Some reports have suggested that DPP-4 inhibition has cardioprotective effects in humans, possibly by enhancing left ventricular functions or myocardial regeneration [49–51]. DPP-4 inhibition improves age-related diseases, such as hypertension, dyslipidemia, hepatic steatosis, and neurodegenerative diseases. DPP-4 inhibition may have a protective effect on cardiovascular diseases, which are a major cause of mortality in patients with diabetes. Because the impact of DPP-4 inhibition on cardiovascular events in diabetic patients likely depends on each patient’s background (i.e., disease duration, age, BMI, current therapy, glycemic control, or complications), further examination of the hypothesis presented in this study in clinical studies focusing on DPP-4 inhibition in humans is required.

## Conclusions

We created a model of nutrient-induced early lethality in an obese diabetic db/db mouse with a diet containing a combination of sucrose and linoleic acid. We also showed that DPP-4 inhibition ameliorated survival in those mice. The results of the current study demonstrate the novel therapeutic potential of DPP-4 inhibitors for the extension of lifespan in diabetes patients.

## Additional files

10.1186/s13098-016-0138-4 Compositions of experimental diets.
10.1186/s13098-016-0138-4 SL-diet-induced early mortality in db/db mice and DPP-4 inhibition reduced lethality. Survival rates of indicated mice in three independent cohort studies. (a) n = 12, (b) n = 8, (c) n = 7. The db/+ and db/db mice were fed the SL diet, SO diet, SL + DPP-4 inhibitor diet, or SO + DPP-4 inhibitor diet, as described in Fig. 2.
10.1186/s13098-016-0138-4 Liver and epididymal fat weights in db/+ mice and db/db mice. The experiments were performed in db/+ or db/db mice fed an SL diet, SO diet, SL containing DPP-4 inhibitor (0.4% des-fluoro-sitagliptin) diet, or SO containing DPP-4 inhibitor diet for 8 weeks. (left) Liver weights as a proportion of body weight (n = 5). (right) Epididymal fat weights as a proportion of body weight (n = 5).
10.1186/s13098-016-0138-4 Biochemical parameters in db/+ mice and db/db mice. Plasma alanine aminotransferase (ALT), free fatty acid (FFA), total cholesterol (TChol), and triglyceride (TG) in the indicated groups of mice (n = 5).
10.1186/s13098-016-0138-4 Liver triglyceride levels in db/+ mice and db/db mice. Concentrations of liver TG (mg/g liver) in the indicated groups of mice (n = 5).
10.1186/s13098-016-0138-4 Cardiac muscle morphology in db/db mice. HE staining (upper) and Masson-Goldner staining (lower) of cardiac muscle in the indicated of mice.
