# Supplementary material for: DPP-4 inhibition improves early mortality, β cell function, and adipose tissue inflammation in db/db mice fed a diet containing sucrose and linoleic acid
Source: Diabetol Metab Syndr. 2016 Mar 1;8:16. doi: 10.1186/s13098-016-0138-4 (PMC4774120; doi:10.1186/s13098-016-0138-4)
Supplement: Supplementary file 1 — 10.1186/s13098-016-0138-4 Compositions of experimental diets. [file 13098_2016_138_MOESM1_ESM.pdf]

Supplementary Table S1. Compositions of experimental diets[a]

	Diet	
	SL	SO
	g/kg diet	
Total milk protein and casein[b]	212.8	212.8
Fat[c]	140.4	140.4
Carbohydrate	510.7	510.7
Indigestible dextrin	63.8	63.8
Vitamin and mineral mixture[d]	72.3	72.3
Fatty acid composition (%)		
Palmitic acid 16:0	7.3	6.7
Stearic acid 18:0	2.6	4.2
Oleic acid 18:1 (n-9)	13.4	72.3
Linoleic acid 18:2 (n-6)	76.4	10.7
α-Linolenic acid 18:3 (n-3)	0.2	4.4
Other fatty acid	0	1.7
Carbohydrate composition, (%)		
Sucrose	70	70
Dextrin	30	30
Total energy, (kJ/g)[e]	17.8	17.8

[a]All the diets were identical except for the type of fat used: linoleic acid was used in the SL diet, and oleic acid was used in the SO diet.

[b]The percentages of total milk protein and casein were 60% and 40%, respectively.

[c]The fat components of SL and SO were derived from safflower oil and high-oleic sunflower oil blended with perilla oil, respectively. The percentages of high-oleic sunflower oil and perilla oil were 90% and 10%, respectively.

[d]The composition of the vitamin and mineral mixture (per kg of diet) was as follows. Vitamins supplied: 3.2 mg retinyl palmitate, 31.9 μg cholecalciferol, 340.4 mg α-tocopherol, 42.6 μg menadione and phylloquinone, 255.3 mg thiamine HCl, 21.3 mg riboflavin, 89.4 mg nicotinamide, 12.8 mg pyridoxine HCl, 42.6 mg calcium pantothenate, 1.7 g L-ascorbic acid, 825.5 mg choline, 2.1 mg folic acid, 38.3 μg cyanocobalamin, and 12.3 μg biotin. Minerals supplied: 3.0 g sodium, 3.4 g calcium, 42.6 mg iron, 3.4 g phosphorus, 1.1 g magnesium, 3.4 g potassium, 2.1 mg copper, 0.4 mg manganese, 42.6 mg zinc, 2.6 g chloride, 148.9 μg selenium, 127.7 μg chromium, 119.1 μg iodine, and 123.4 μg molybdenum.

[e]The percentages of energy as fat, carbohydrate, and protein were 29.7%, 50.3%, and 20.0%, respectively.
