# Supplementary material for: DPP-4 inhibition improves early mortality, β cell function, and adipose tissue inflammation in db/db mice fed a diet containing sucrose and linoleic acid
Source: Diabetol Metab Syndr. 2016 Mar 1;8:16. doi: 10.1186/s13098-016-0138-4 (PMC4774120; doi:10.1186/s13098-016-0138-4)
Supplement: Supplementary file 2 — 10.1186/s13098-016-0138-4 SL-diet-induced early mortality in db/db mice and DPP-4 inhibition reduced lethality. Survival rates of indicated mice in three independent cohort studies. (a) n = 12, (b) n = 8, (c) n = 7. The db/+ and db/db mice were fed the SL diet, SO diet, SL + DPP-4 inhibitor diet, or SO + DPP-4 inhibitor diet, as described in Fig. 2. [file 13098_2016_138_MOESM2_ESM.pdf]

Supplementary Figure S1

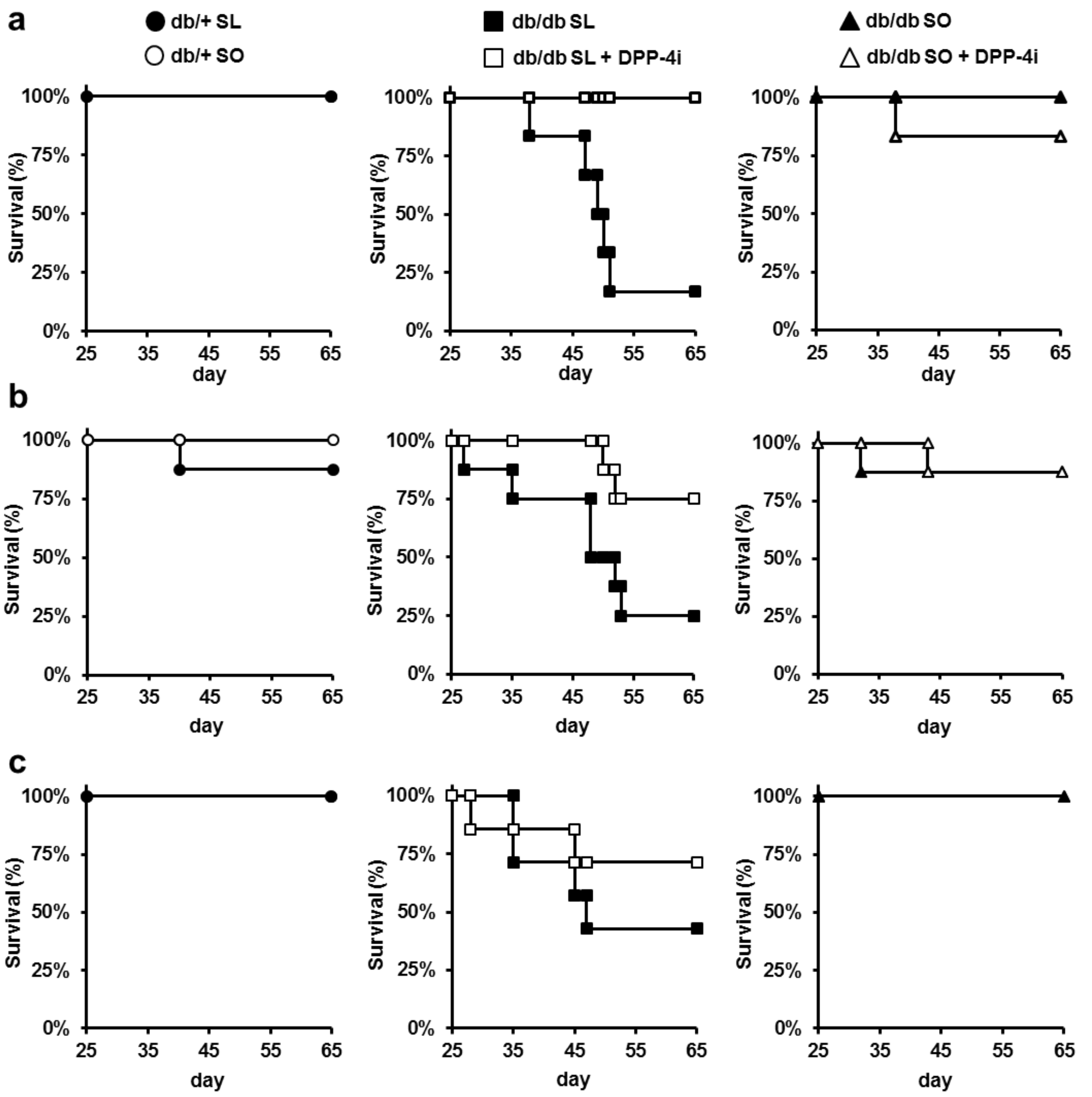

Supplementary Figure S1. SL-diet-induced early mortality in db/db mice and DPP-4 inhibition reduced lethality.

Survival rates of indicated mice in three independent cohort studies. (a) n = 12, (b) n = 8, (c) n = 7. The db/+ and db/db mice were fed the SL diet, SO diet, SL + DPP-4 inhibitor diet, or SO + DPP-4 inhibitor diet, as described in Fig. 2.
