# Supplementary material for: DPP-4 inhibition improves early mortality, β cell function, and adipose tissue inflammation in db/db mice fed a diet containing sucrose and linoleic acid
Source: Diabetol Metab Syndr. 2016 Mar 1;8:16. doi: 10.1186/s13098-016-0138-4 (PMC4774120; doi:10.1186/s13098-016-0138-4)
Supplement: Supplementary file 3 — 10.1186/s13098-016-0138-4 Liver and epididymal fat weights in db/+ mice and db/db mice. The experiments were performed in db/+ or db/db mice fed an SL diet, SO diet, SL containing DPP-4 inhibitor (0.4% des-fluoro-sitagliptin) diet, or SO containing DPP-4 inhibitor diet for 8 weeks. (left) Liver weights as a proportion of body weight (n = 5). (right) Epididymal fat weights as a proportion of body weight (n = 5). [file 13098_2016_138_MOESM3_ESM.pdf]

Supplementary Figure S2

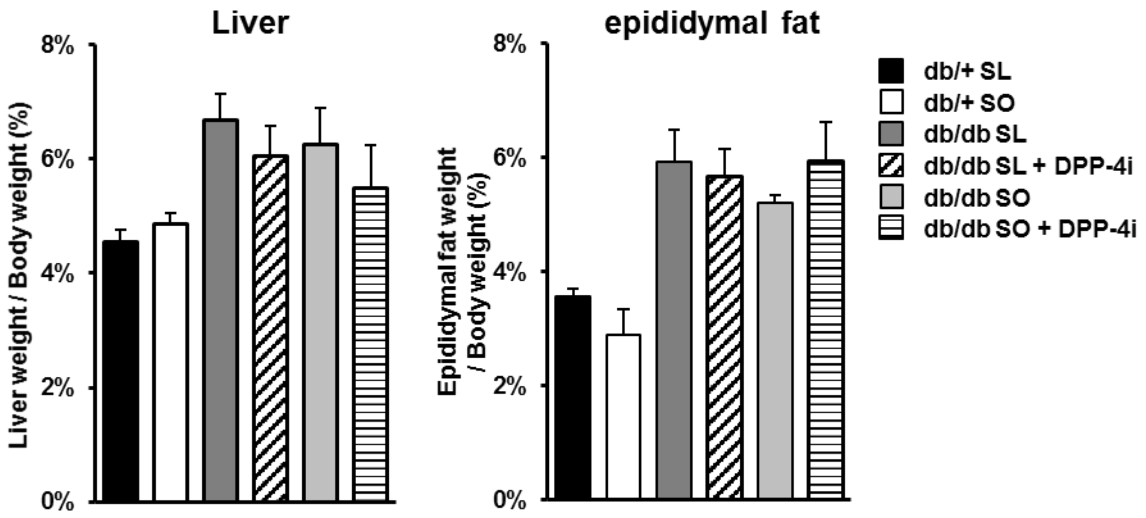

Supplementary Figure S2. Liver and epididymal fat weights in db/+ mice and db/db mice. The experiments were performed in db/+ or db/db mice fed an SL diet, SO diet, SL containing DPP-4 inhibitor (0.4% des-fluoro-sitagliptin) diet, or SO containing DPP-4 inhibitor diet for 8 weeks. (left) Liver weights as a proportion of body weight (n = 5). (right) Epididymal fat weights as a proportion of body weight (n = 5).
