# Supplementary material for: DPP-4 inhibition improves early mortality, β cell function, and adipose tissue inflammation in db/db mice fed a diet containing sucrose and linoleic acid
Source: Diabetol Metab Syndr. 2016 Mar 1;8:16. doi: 10.1186/s13098-016-0138-4 (PMC4774120; doi:10.1186/s13098-016-0138-4)
Supplement: Supplementary file 4 — 10.1186/s13098-016-0138-4 Biochemical parameters in db/+ mice and db/db mice. Plasma alanine aminotransferase (ALT), free fatty acid (FFA), total cholesterol (TChol), and triglyceride (TG) in the indicated groups of mice (n = 5). [file 13098_2016_138_MOESM4_ESM.pdf]

**Supplementary Figure S3**

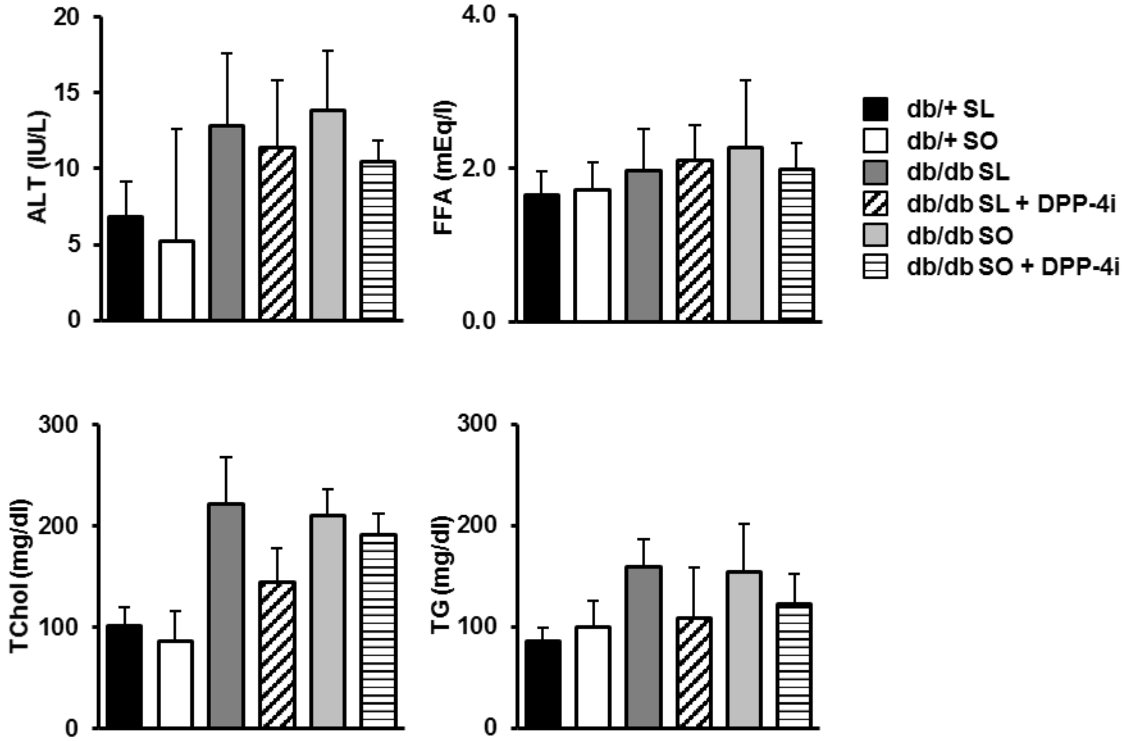


**Supplementary Figure S3. Biochemical parameters in db/+ mice and db/db mice.**

Plasma alanine aminotransferase (ALT), free fatty acid (FFA), total cholesterol (TChol), and triglyceride (TG) in the indicated groups of mice (n = 5).
