# Supplementary material for: DPP-4 inhibition improves early mortality, β cell function, and adipose tissue inflammation in db/db mice fed a diet containing sucrose and linoleic acid
Source: Diabetol Metab Syndr. 2016 Mar 1;8:16. doi: 10.1186/s13098-016-0138-4 (PMC4774120; doi:10.1186/s13098-016-0138-4)
Supplement: Supplementary file 6 — 10.1186/s13098-016-0138-4 Cardiac muscle morphology in db/db mice. HE staining (upper) and Masson-Goldner staining (lower) of cardiac muscle in the indicated of mice. [file 13098_2016_138_MOESM6_ESM.pdf]

Supplementary Figure S5

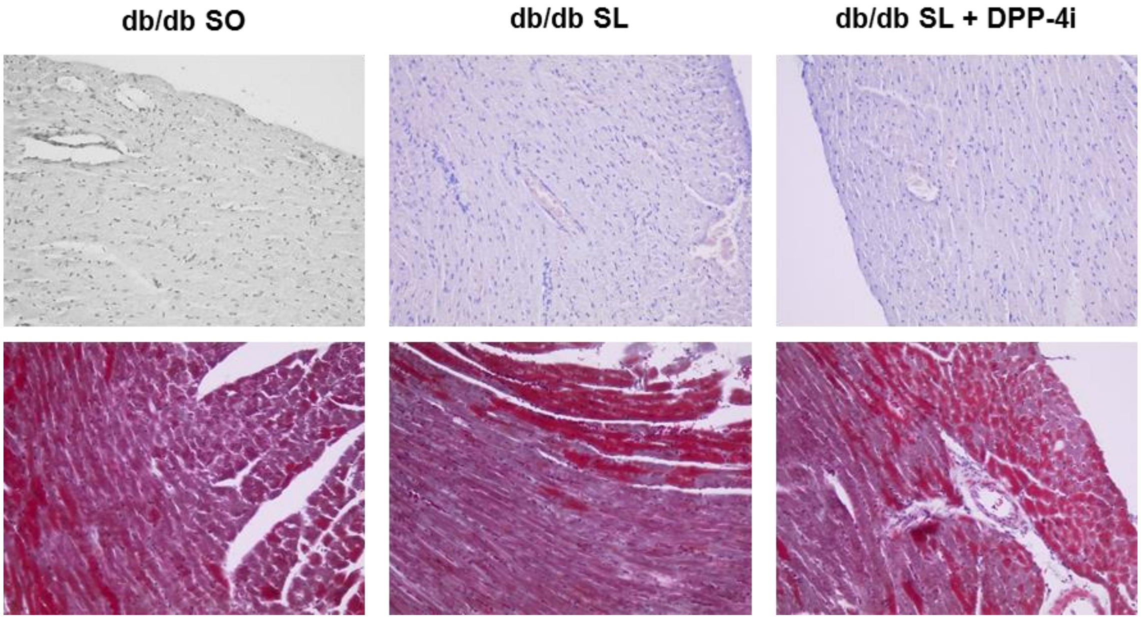

Supplementary Figure S5. Cardiac muscle morphology in db/db mice.

HE staining (upper) and Masson-Goldner staining (lower) of cardiac muscle in the indicated of mice.
